# Impact of Coronavirus disease (COVID-19) pandemic on health professionals

**DOI:** 10.12669/pjms.36.COVID19-S4.2779

**Published:** 2020-05

**Authors:** Bilal Ahmed Sethi, Ahsan Sethi, Sadaf Ali, Hira Shireen Aamir

**Affiliations:** 1 Bilal Ahmed Sethi, Associate Professor and Consultant, Department of Pediatrics, Northwest General Hospital and Research Centre, Peshawar, Pakistan; 2 Ahsan Sethi, Assistant Professor, Khyber Medical University, Peshawar - Pakistan; 3 Sadaf Ali, Lecturer, Khyber Medical College, Peshawar - Pakistan; 4 Hira Shireen Aamir, Medical Resident, Khyber Teaching Hospital, Peshawar - Pakistan

**Keywords:** Challenges, COVID-19, Disease Outbreaks, Health Professionals

## Abstract

**Objective:**

Recognizing the huge potential ramifications of COVID-19 pandemic, this study explores its impact on health professionals personally and professionally along with the associated challenges.

**Methods:**

A descriptive cross-sectional qualitative survey was conducted from March-April 2020. Participants included health professionals from various disciplines in both public and private-sector institutions of Pakistan. The sample size was not predetermined, and an iterative approach of simultaneous data collection and analysis was taken until data and time saturation were reached. Thematic analysis of the qualitative data was carried out by two analysts.

**Results:**

Two hundred and Ninety health professionals responded. They reported an impact on their mental, physical and social well-being. The clinicians mentioned facing an unprecedented workload in overstretched health facilities, while those in academia become engaged with planning/providing emergency remote teaching for the students affecting work-life balance. Some challenges associated with work-from-home and in the hospitals were identified.

**Conclusion:**

During COVID-19, the health professionals are anxious, overworked and financially unstable while planning, creating and caring for others and their families. We need to support them to do their jobs, be safe and stay alive. Future research should explore the fears and coping strategies of health professionals during pandemics.

## INTRODUCTION

Coronavirus disease (COVID-19) caused by severe acute respiratory syndrome coronavirus 2 (SARS-CoV-2) is an infectious disease.^1^ After its outbreak in Wuhan, China (December 2019), the rapid spread of the virus sparked alarm worldwide. The World Health Organization declared this outbreak a pandemic, and countries around the world are grappling with a surge in confirmed cases. As of May 4, 2020, over 3.3 million confirmed cases of COVID-19 have been reported, resulting in more than 238,730 deaths in 215 countries.^2^ As the virus spreads through close contact and by small droplets produced during cough, sneeze or talk,^1^,^3^ most of countries have responded with preventive measures through health advocacy campaigns, lockdowns and restricting public gatherings. The hospitals are ramping up their capabilities to care for increasing numbers of infected patients. Meanwhile, scientists are exploring potential treatments, testing new therapies and vaccines to contain the virus.^4^

In Pakistan the situation is no different with 20,186 confirmed cases of COVID-19 and 462 deaths till date.^5^ To control the spread of COVID-19, the government has implemented partial lockdowns with complete closure of non-essential services, shops, restaurants and air travel. However, an across-the-board enforcement of such lockdowns remains beyond the capacity of the state and the numbers of people testing positive to COVID-19 continue to rise.

To strengthen country and community emergency preparedness, the Government issued directives for closure of OPDs and elective surgical services in all the Tertiary Care Hospitals, District Headquarters Hospitals and Private Clinics.^6^ Specific hospitals have been designated for screening and management of cases. Other measures included establishment of quarantine centers, testing facilities and public awareness regarding COVID-19.^7^ All the academic institutions were also given a three-week break. Recently, the Higher Education Commission (HEC), Pakistan issued guidelines suggesting delivery of online courses to minimize academic loss.^8^

The health professionals have to juggle multiple roles including administrative, clinical and educational roles.^9^ During COVID-19 pandemic, the added online educational and clinical responsibilities may have had an impact. Little previous history, evidence-base and lack of vaccine to contain this novel virus may have also incited panic. Recognizing the huge potential ramifications, this study explores the impact of COVID-19 pandemic on health professionals personally and professionally, along with associated challenges.

## METHODS

This descriptive cross-sectional survey was conducted from March-April 2020. Ethical approval was granted by Northwest General Hospital and Research Centre, Peshawar.

### Questionnaire

An open-ended qualitative questionnaire was developed, asking the participants regarding the personal and professional impact of COVID-19, and the associated challenges. The questionnaire was piloted (n=12) to check for comprehension, accessibility and technical compatibility. All the items were kept mandatory.

### Data Collection

Sampling frame included health professionals from various disciplines in both public/private medical and dental institutions in Pakistan. The questionnaire was shared through email, virtual learning environments and snowballing via social networks (e.g. WhatsApp and Facebook groups). The sample size was not predetermined, and an iterative approach of simultaneous data collection and analysis was taken until data and time saturation were reached.

### Data Analysis

Descriptive statistics were calculated for the demographic data. For qualitative data, each response was carefully read to develop in-vivo analytic codes. The selective codes were categorized, and themes were developed.^10^ Through reflective thinking and team discussions the themes were continuously refined to fit the data.

## RESULTS

Two hundred and Ninety health professionals at varying stages in their professional careers and from both public and private-sector institutions of Pakistan responded. The respondents were predominantly female (56.5%) and from age range 36-55 years (65.5%) (Table-I).

**Table-I:** Participant Characteristics.

Characteristics			N(%)
Gender	Male		126(43.45)
	Female		164(56.55)
Age	26-35 Yrs.		72(24.83)
	36-45 Yrs.		104(35.86)
	46-55 Yrs.		86(29.65)
	56-65 Yrs.		28(9.66)
Workplace	Punjab		157(54.14)
	Khyber Pakhtunkhwa		92(31.72)
	Sindh		41(14.14)
Discipline	Medicine	Basic Sciences	71(24.48)
		Clinical Sciences	166(57.24)
	Dentistry		35(12.07)
	Medical Education		18(6.21)

### Personal impact

The health professionals reported an impact on their mental, physical and social well-being. They felt anxious, frustrated and stressed out. Their social life and other routine activities were disrupted due to travel restrictions. They were not able to attend funerals and many gatherings were postponed. Some reported difficulty in coping up with the required standards and needs of social distancing during this pandemic. A few reported having more time for self, family and work (Table-II).

**Table-II:** Impact of COVID-19 on health professionals.

Personal	Health	Mental	Psychological battle with uncertainty. I’m in state of fear and stress and sometimes depression too…I cannot sleep at night
		Physical	Lot of weight gain as no physical activity with eating foods all day
		Social	It has changed my social, family and domestic life. I am avoiding unnecessary travel…unnecessary gatherings…hugging family
	More time to spare	Self	It has given me time to reflect on things about myself…which I didn’t have time to think about before…I started regular exercise…and reading
		Family	I feel myself lucky to let my daughter offer prayers with me at a very little age (7.5Yrs.) and I am able to make her learn how to recite Quran Pak. In past I could not get that much time to spend home with kids
		Work	Worked on a couple of research papers
Professional	Temporary layoff	Academicians Clinicians	The University is closed, Academics suspended. Evening Clinical Practice is also closed. We have to postponed elective cases till further orders…Even for emergency cases, being a private-sector hospital, we have been asked to send the patients to government hospitals. So basically, we are not ‘treating’ our patients anymore
	Increased workload	Academicians Clinicians	Unable to teach or deliver lectures at workplace so working from home and online. Engaged 24/7 while fighting against COVID-19. Social and family life is too much affected…emergency duties in the hospital as well as in the field
	Offering remote access and services	Academicians Clinicians	As medical teachers, we are conducting online classes. I record my audio/video lecture on zoom and upload it on google classroom. Patients don’t have access to us & we have started Tele-Clinic for them
	Financial instability	Pay cut	Pay cut imposed by our institution is disheartening, even though we are working from home and working 24/7
		No private practice	Clinic closed. All clinic staff restrained to their homes, but I am paying them from my pocket which is very difficult
	Learning activities	Postgraduate courses	Academically our classes of Masters’ session postponed, so we might get late in our academic years.
		Postgraduate exams	Having lined up exams being cancelled indefinitely
		Continuing Medical Education	Two important seminars missed, one in Riyadh Saudi heart Association and another ACC in Chicago.
		Research and Scholarship	All my research projects and funded projects are stopped because of social distancing.
	Promotions		I am due for my professorship but due to this uncertainty my application is stuck

### Professional impact

Health professionals experienced a temporary layoff initially with closure of academic institutions and private clinics, and disruption of hospital OPDs, elective procedures. However, their roles and responsibilities increased over the period of time. The clinicians mentioned facing an unprecedented workload in overstretched health facilities, while those in academia become engaged with planning/providing emergency remote teaching for the students. Many participants offered remote access through telephone to their patients. The financial fallout of the pandemic also triggered an outcry from those working in private-sector or running private clinics. They reported experiencing pay cuts and reduced income, while also supporting household/clinic workers. Some had an impact on their learning and promotions (Table-II).

### Challenges

Health professionals reported various challenges related to work from home and in the hospital. They found it hard to work and also manage their household, families and children. The household helpers were also not there to support. They were not satisfied with the effectiveness of online teaching and mentioned their lack of training. In the hospital, the participants found it challenging to ensure their safety. They reported unrealistic expectations to manage the patients without any safety provisions. The standards observed in the hospital were far less than that needed for tackling COVID-19. They found it hard to stay motivated. Indifferent attitude of the public due to paranoia, misinformation and non-compliance was also challenging (Table-III).

**Table-III:** Challenges associated with COVID-19.

Working from home	Managing Home and Family	No helping hand	Difficult to manage all my professional work at home with all the house chores. I have given off to all maids so have to manage cleaning, washing, cooking, kids’ online classes and my online lectures.
		Engaging children	Kids at home. School off. They want constant attention.
	Emergency remote learning	Lack of satisfaction	We are conducting online classes. I record my audio/video lecture on zoom and upload it on google classroom. The satisfaction that your students have understood the topic is not possible this way.
		Lack of training	Most of the faculty members are technophobes. It becomes a huge challenge to get through a day’s work. Faculty is not trained for online teaching
		Resistance	Resistance from student and faculty side for online teaching
		Accessibility	Restriction to coordinate with others, those not that tech savvy, those stuck in remote areas, net issues
		Limitations	Difficult for students to understand complex concepts. Lack of student one-to-one interaction. Practical sessions cannot be conducted. Students assessments/exams are affected.
Working in the hospital	Ensuring Safety	Unrealistic expectations	Demands by Hospital Administration to work with patients without appropriate safety kits
		Lack of standard operating procedures	Have to work in an uncertain situation…No SOP available to manage patients…Patient satisfaction and treatment modalities are not up to standard
		Lack of resources	We have very little resources and too much constraints in dealing with this dreadful situation
	Keeping staff motivated		Keeping your healthcare workers motivated
	Breaking Bad News		It was very challenging to break the news of positive corona PCR result.
	Indifferent attitude of public	Paranoia	Lack of correct awareness in public. Too many paranoid people requesting for testing
		Ignorance	Various rumours & fake notifications have made condition worse. One rumour was that government has ordered to kill corona positive patients & their close contacts, so people even with contact history or symptoms were hiding
		Non-compliance	Public in general is very careless…they do not understand the importance of social distancing…their carelessness can affect the people who care.

## DISCUSSION

Since the last pandemic, our understanding of disease transmission and management has improved. However, COVID-19 has shown the limits of our ability. This study explored the impact of COVID-19 pandemic on the health professionals in Pakistan. The study found an impact on the physical, mental and social well-being of health professionals as also reported from china.^11^ They felt considerable mental stress as they too have families, and so will also naturally be fearful that the virus might reach them. Institutions should ensure that health professionals have access to counselling services, so that they can continuously recharge, given that this battle could go long. Many clinicians experienced increased responsibilities related to COVID-19 in screening areas, testing labs, isolation wards and quarantine centers as also highlighted by Waris et al.^7^ They reported stepping directly into COVID-19’s path to aid the affected people and help halt the spread. The day-night work of health professionals have been recognized in other countries as well with many health professionals being infected and died.^12^

As private clinics were closed, some doctors adopted telemedicine to reach their patients. This may be because currently health professionals have more usage of technology and internet on day-to-day basis for research and entertainment purposes.^13^ It may also be related to their thirst for learning new methods to match with the evolving requirements of healthcare and education industry globally. To avoid possible risk of infection, those in academia were also asked to work from home. They adopted various online learning tools for teaching and learning, giving synchronous and asynchronous lectures, and also uploading resource materials.

The current study identified various challenges related to work-from-home and in the hospital. The technology has blurred the boundaries between work and home life^14^ as they were managing the office work, household and taking care of their families, all at the same time. With nowhere to go, they felt like they have no legitimate excuse for being unavailable. We recommend that work from home should not mean work 24/7 and they should create routines, have designated space and avoid extreme multitasking.^15^ Limitations such as inability to teach skills can be managed through procedural videos, synchronous demonstrations, 3D/virtual learning technologies and second-life for teaching technical skills.^16^ To improve satisfaction, it is important that the course designers offer authentic learning experiences with active participation for engagement and deeper learning.^17^ Faculty development in online modalities and lesson planning is recommended.

Those working in the hospital reported their vulnerability to getting infected due to lack of personal protective equipment, standard operating procedures and infrastructure. We recommend telephone triage to prevent patients who can be cared for at home from coming to hospital.^18^ Health professionals dealing with COVID-19 should have separately assigned rooms to wear/remove scrubs. They should be given protective gears such as gowns, face-masks, glasses and face-shields.^7^ Once off duty, they should fully decontaminate themselves to prevent infection to themselves and their families. The public should be considerate of health professionals and the limitations of healthcare system. As misinformation can cause public panic and unrest, they should be asked to consider using government or hospital website/social media pages/mobile applications for COVID-19 related updates.^18^

Our study participants mostly belonged to the medical colleges of Punjab and Khyber Pakhtunkhwa with fewer participants from Sindh province. Yet the findings offer an understanding of the impact of COVID-19 on health professional and identified the associated challenges from diverse range of health professionals. Due to the novelty of COVID-19, there is a lack of similar previously published studies, therefore comparison of all aspects of the results was difficult.

## CONCLUSION

During COVID-19, the health professionals are anxious, overworked and financially unstable. Despite the challenges, they are working, planning, creating and caring for others and their families. Their heroism, dedication and selflessness offers reassurance that we will be able to overcome this virus. We need to give them all the support they need to do their jobs, be safe and stay alive. Future research should explore the fears and coping strategies of health professionals as frontline soldiers during pandemics.

### Authors’ Contribution

**BAS and AS** conceived the idea and designed the study.

All the authors (**BAS, AS, SA and HAS)** were involved in data collection, analysis and data interpretation. All contributed towards writing the manuscript, approved the final version and are accountable.
